# Supplementary material for: High-severity wildfires in temperate Australian forests have increased in extent and aggregation in recent decades
Source: PLoS One. 2020 Nov 18;15(11):e0242484. doi: 10.1371/journal.pone.0242484 (PMC7673578; doi:10.1371/journal.pone.0242484)
Supplement: S1 File — (DOCX) [file pone.0242484.s001.docx]

**S1 Table.** Description of landscape metrics.

| **High-severity Area** | | | | | |
| --- | --- | --- | --- | --- | --- |
| $\sum_{i=1}^{n} a_{i}$ | | | | a_i_ =   area (ha) of high-severity patch i. | |
| *Description* | Equals the sum of the areas (ha) of all high-severity patches | | | | |
| *Units* | Hectares | | | | |
| *Range* | > 0, without limit. | | | | |
| **Percentage of High-severity Area** | | | | | |
| $\frac{\sum_{i=1}^{n} a_{i}}{A} \left( 100 \right)$ | | | | a_i_ =     area (ha) of high-severity patch i.  A =     total fire area (ha). | |
| *Description* | The proportion of the total fire area (all fire severities) occupied by high-severity patches equals the sum of the areas (ha) of all high-severity patches, divided by total fire area (ha), multiplied by 100 (to convert to a percentage) | | | | |
| *Units* | Percent | | | | |
| *Range* | (0-100) | | | | |
| **Patch size- Mean Area** | | | | | |
| $\frac{\sum_{i=1}^{n} a_{i}}{n_{i}}$ | | | | a_i_ =     area (ha) of high-severity patch i.  n_i_ =     number of high-severity patches. | |
| *Description* | The mean patch size area equals the sum area, across all high-severity patches, divided by the number of high-severity patches. | | | | |
| *Units* | Hectares | | | | |
| *Range* | > 0, without limit. | | | | |
| **Patch size – Coefficient of variation** | | | | | |
| $\frac{SD}{Mean} \left( 100 \right)$ | | | | _SD_ =     Standard deviation of high-severity patch size.  Mean =     Mean high-severity patch size. | |
| *Description* | CV (coefficient of variation) equals the standard deviation of patch size divided by the mean patch size, multiplied by 100 to convert to a percentage, for high-severity patches. | | | | |
| *Units* | Percent | | | | |
| *Range* | (0-100) | | | | |
| **Number of Patches** | | | | | |
| $\left( n \right)$ | | | n =     number of high-severity patches. | | |
| *Description* | It equals the number of high-severity patches within the total fire area | | | | |
| *Units* | None | | | | |
| *Range* | ≥ 1, without limit. | | | | |
| **Edge Density** | | | | | |
| $\frac{\sum_{i=1}^{n} e_{i}}{A}$ | | | | ei =    total length (m) of edge of high-severity patches  A =     total fire area (ha). | |
| *Description* | It equals the sum of the lengths (m) of all edge segments involving high-severity patches, divided by total fire area (ha). | | | | |
| *Units* | Meters per hectare | | | | |
| *Range* | ≥ 0, without limit. | | | | |
| **Clumpiness** | | | | | |
| 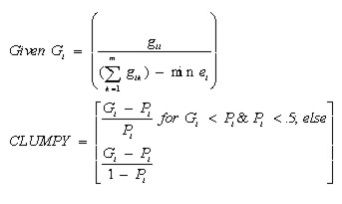 | | | | | g_ii_ =    number of like adjacencies (joins) between high-severity pixels based on the *double-count* method.  g_ik_ = number of adjacencies (joins) between high-severity pixels based on the *double-count* method.  min-e_i_ = minimum perimeter (in number of cell surfaces) of high-severity pixels for a maximally clumped class.  P_i_ = proportion of the total fire area occupied by high-severity pixels |
| *Description* | It equals the proportional deviation of the proportion of like adjacencies involving high-severity pixels from that expected under a spatially random distribution. Cell adjacencies are tallied using the *double-count* method in which pixel order is preserved. | | | | |
| *Units* | None | | | | |
| *Range* | (-1, 1)  Clumpiness equals -1 when the focal patch type is maximally disaggregated; Clumpiness equals 0 when the focal patch type is distributed randomly and approaches 1 when the patch type is maximally aggregated. | | | | |
| **Normalized Landscape Shape Index** | | | | | |
| 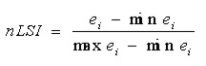 | | | | e_i=_ total length of edge (or perimeter) of high-severity in terms of number of cell surfaces  min e_i_ = minimum total length of edge (or perimeter) of high-severity in terms of number of cell surfaces  max e_i_ =   maximum total length of edge (or perimeter) of high-severity in terms of number of cell surfaces. | |
| *Description* | | It equals the total length of edge (or perimeter) involving the high-severity class, given in number of cell surfaces, minus the minimum length of class edge (or perimeter) possible for a maximally aggregated class, also given in number of cell surfaces, which is achieved when the class is maximally clumped into a single, compact patch, divided by the maximum minus the minimum length of class edge. | | | |
| *Units* | | None | | | |
| *Range* | | (0 – 1)  NLSI = 0 when the landscape consists of a single square or maximally compact (i.e., almost square) patch of the corresponding type; NLSI increases as the patch type becomes increasingly disaggregated and is 1 when the patch type is maximally disaggregated (i.e., a checkerboard when P_i_ ≤ 0.5). | | | |

**S2 Table.** Pearson’s correlation among landscape metrics characterizing the extent and spatial configuration of areas burned by high-severity fire. Variables were natural log (Area, Number of Patches, Mean Patch Area, Variation Patch Area, NLSI) or arcsine (Proportional area, Edge Density) transformed.

|  | **High-severity burnt area** | **Proportional**  **area** | **Number of Patches** | **Edge**  **Density** | **Mean Patch Area** | **Variation Patch Area^a^** | **Clumpiness** | **NLSI^b^** |
| --- | --- | --- | --- | --- | --- | --- | --- | --- |
| High-severity burnt area | 1.00 |  |  |  |  |  |  |  |
| Proportional area | 0.68 | 1.00 |  |  |  |  |  |  |
| Number of Patches | 0.61 | -0.11 | 1.00 |  |  |  |  |  |
| Edge Density | 0.54 | 0.61 | 0.25 | 1.00 |  |  |  |  |
| Mean Patch Area | 0.58 | 0.88 | -0.25 | 0.32 | 1.00 |  |  |  |
| Variation Patch Area | 0.86 | 0.47 | 0.66 | 0.57 | 0.35 | 1.00 |  |  |
| Clumpiness | 0.51 | 0.35 | 0.13 | 0.25 | 0.37 | 0.59 | 1.00 |  |
| NLSI | -0.72 | -0.87 | -0.04 | -0.58 | -0.82 | -0.63 | -0.51 | 1.00 |

^a^ Variation Patch area calculated as Coefficient of Variation of Patch Area

^b^  NLSI: Normalised Landscape Shape Index (Turner et al., 2001)

**
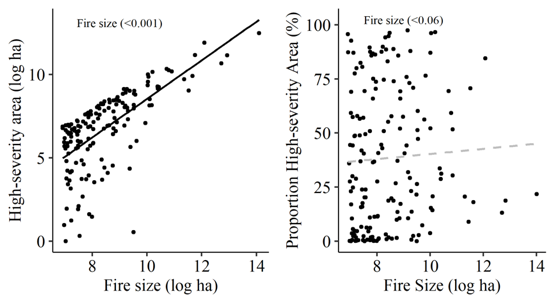
**

**S1 Fig**. Relationship between total and proportional high-severity burned area with fire size (i.e. total fire area). Values for 162 wildfires between 1987 and 2017.

**
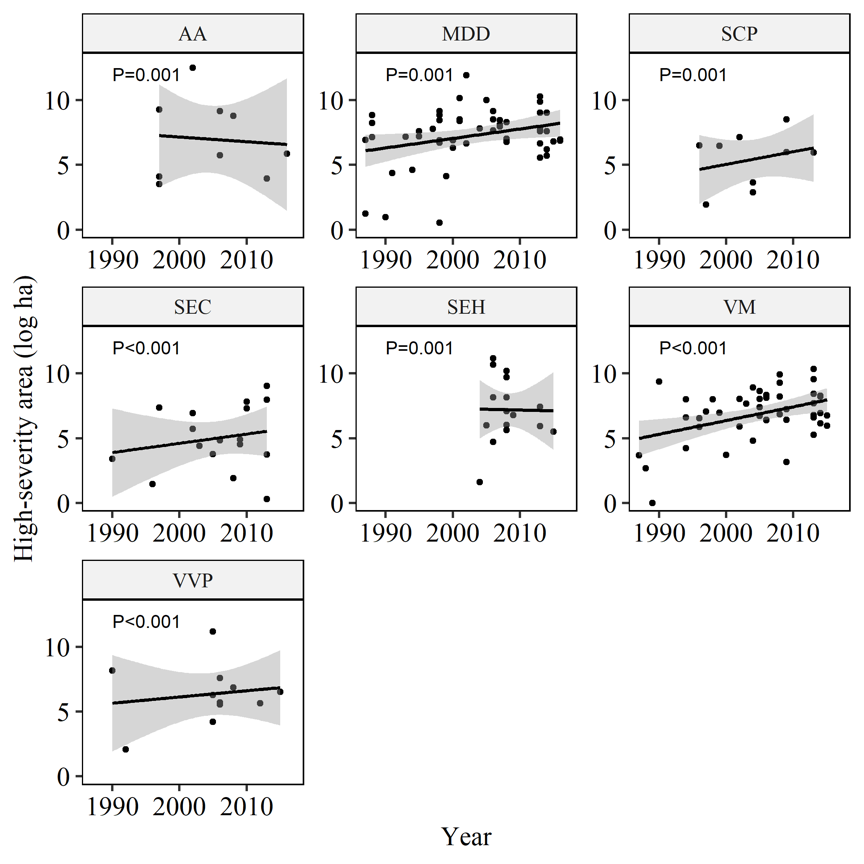
**

**S2 Fig**. Changes in high-severity burned area per year from 1987 to 2017 across studied bioregions. Acronyms follow Table 1.


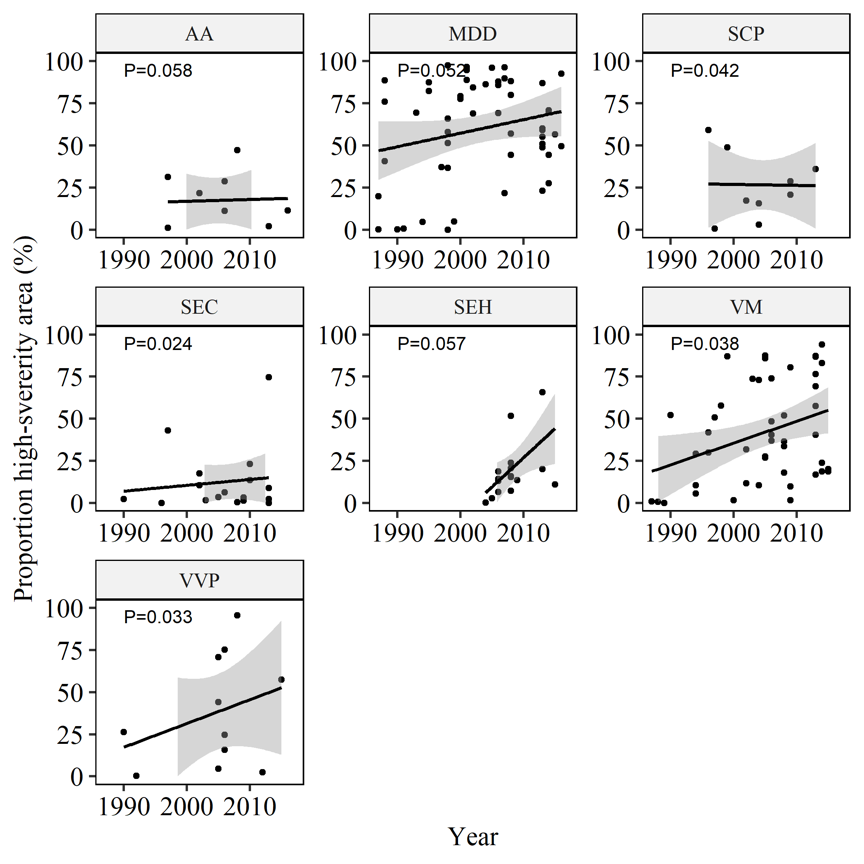


**S3 Fig**. Changes in proportional high-severity burned area per year from 1987 to 2017 across bioregions. Acronyms follow Table 1.


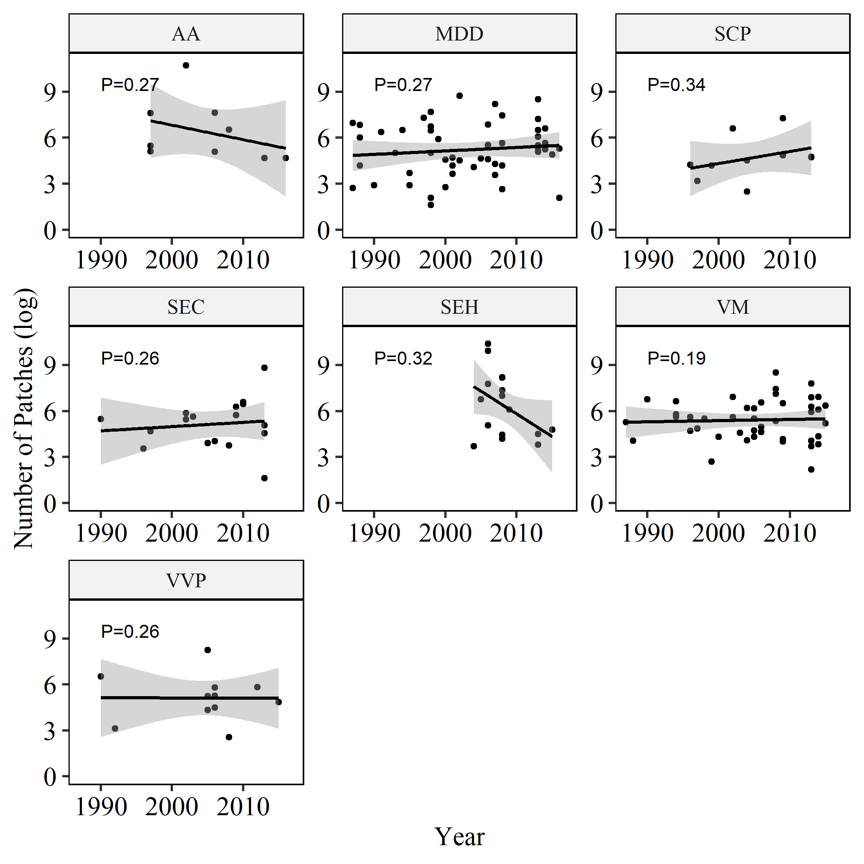
**S4 Fig**. Changes in the number of high-severity patches per year from 1987 to 2017 across bioregions. Acronyms follow Table 1.

**
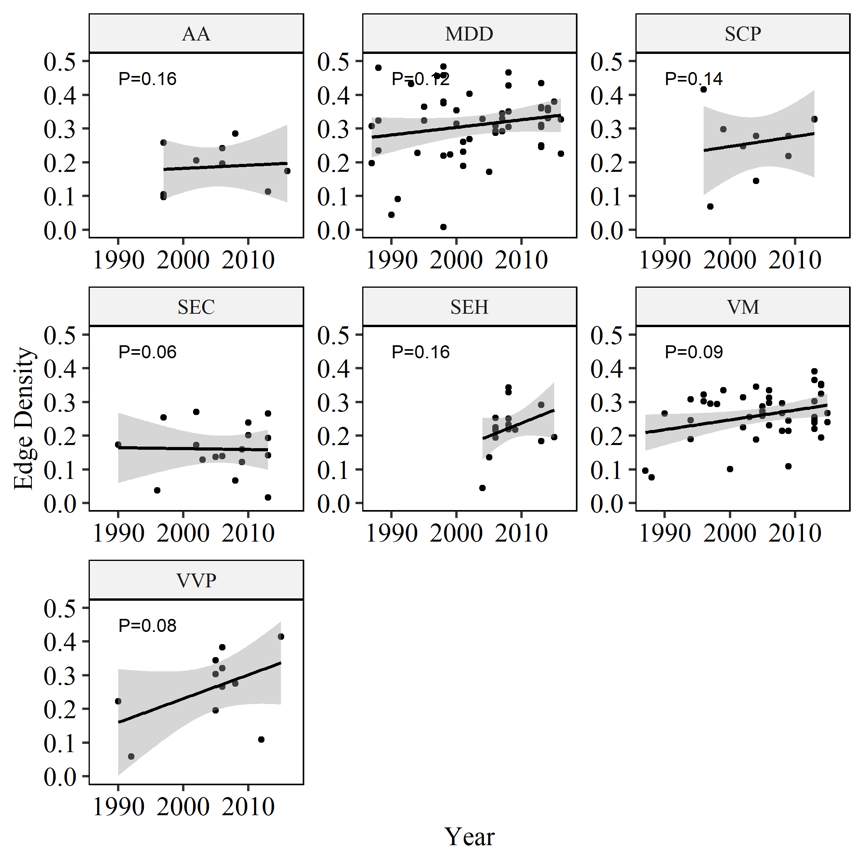
S5 Fig**. Changes in the edge density of high-severity patches per year from 1987 to 2017 across bioregions. Acronyms follow Table 1.


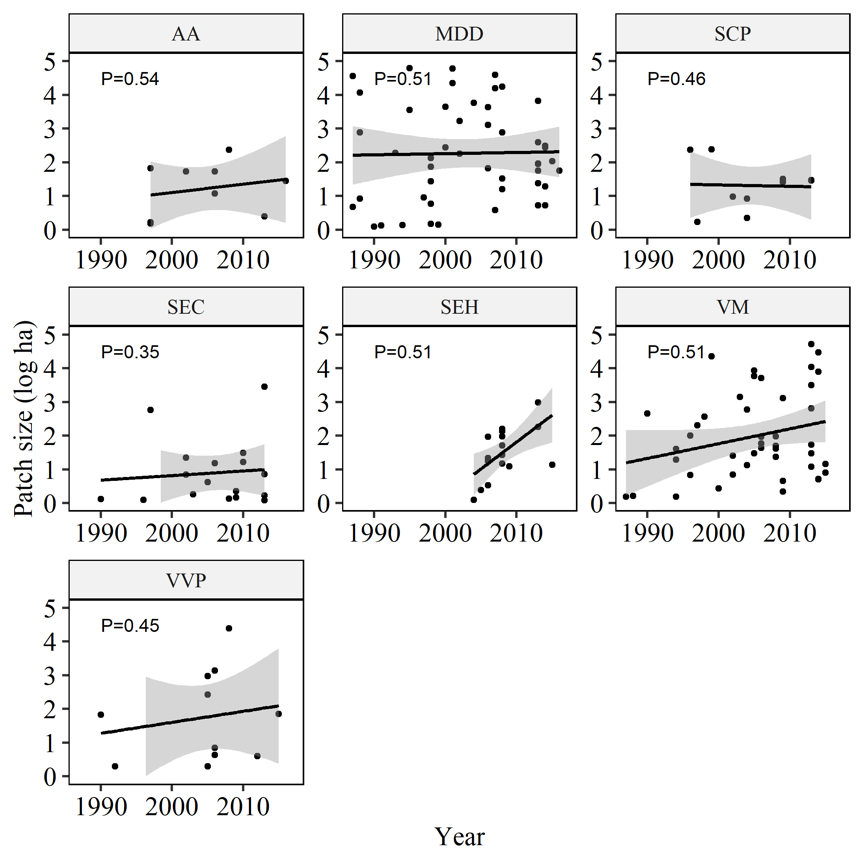
**S6 Fig.** Changes in the mean high-severity patch size per year from 1987 to 2017 across bioregions. Acronyms follow Table 1.

**
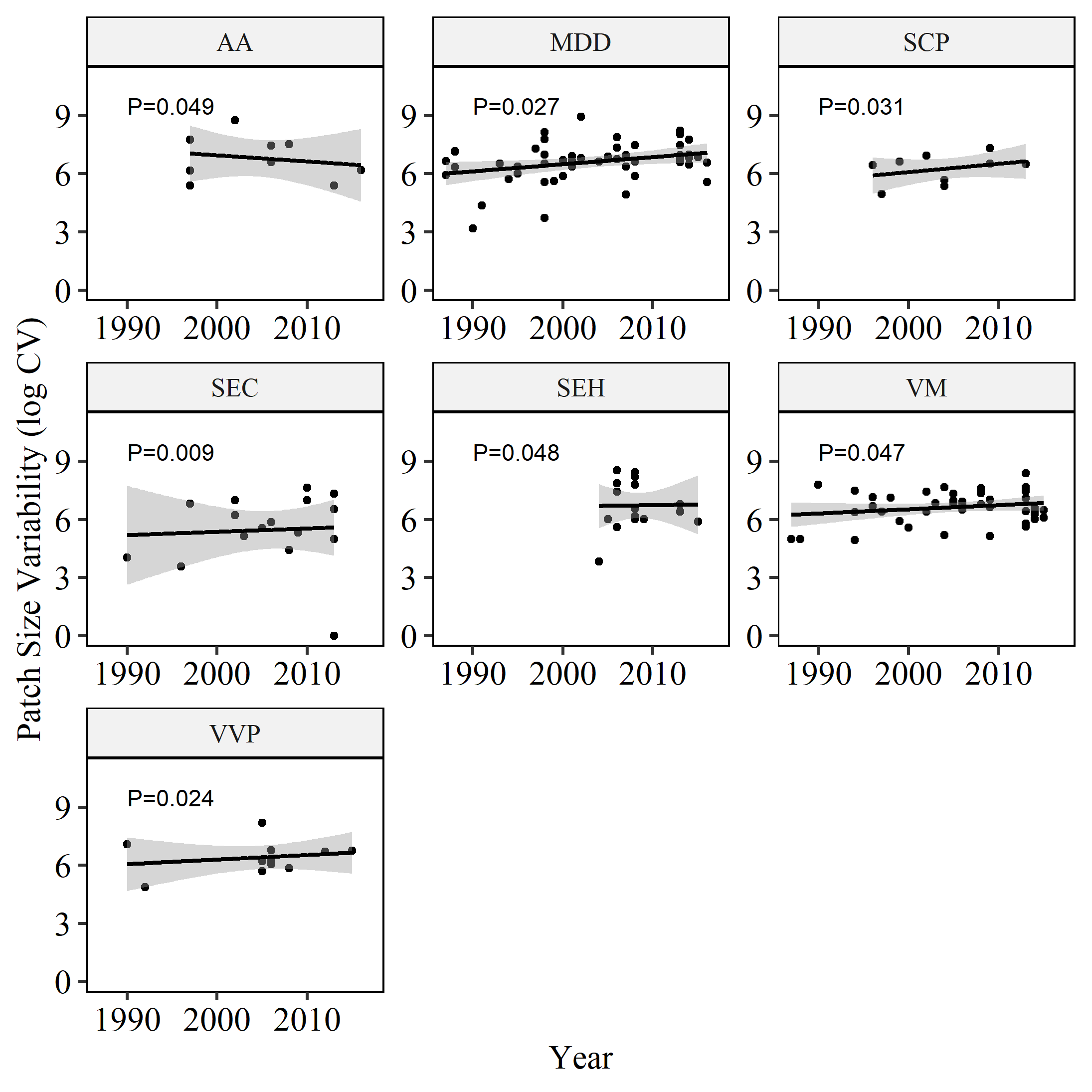
S7 Fig**. Changes in the coefficient of variation of high-severity patch size per year from 1987 to 2017 across bioregions. Acronyms follow Table 1.

**
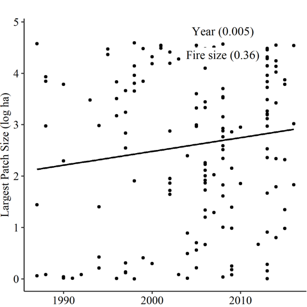
**

**S8 Fig**. Changes in the size of the largest high-severity patch per year from 1987 to 2017 in the state of Victoria, Australia.

**
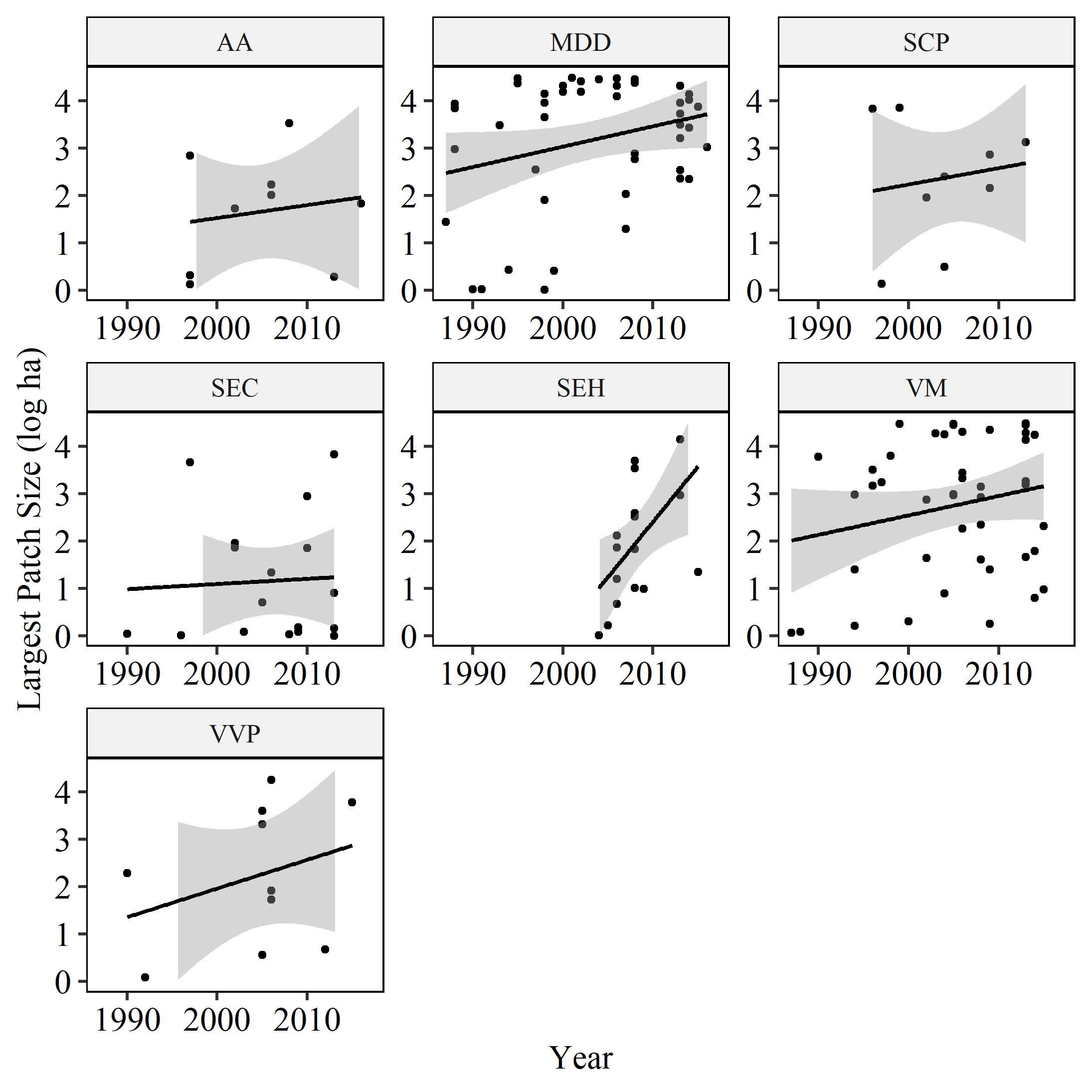
**

**S9 Fig**. Changes in the size of the largest high-severity patch per year from 1987 to 2017 across bioregions. Acronyms follow Table 1.


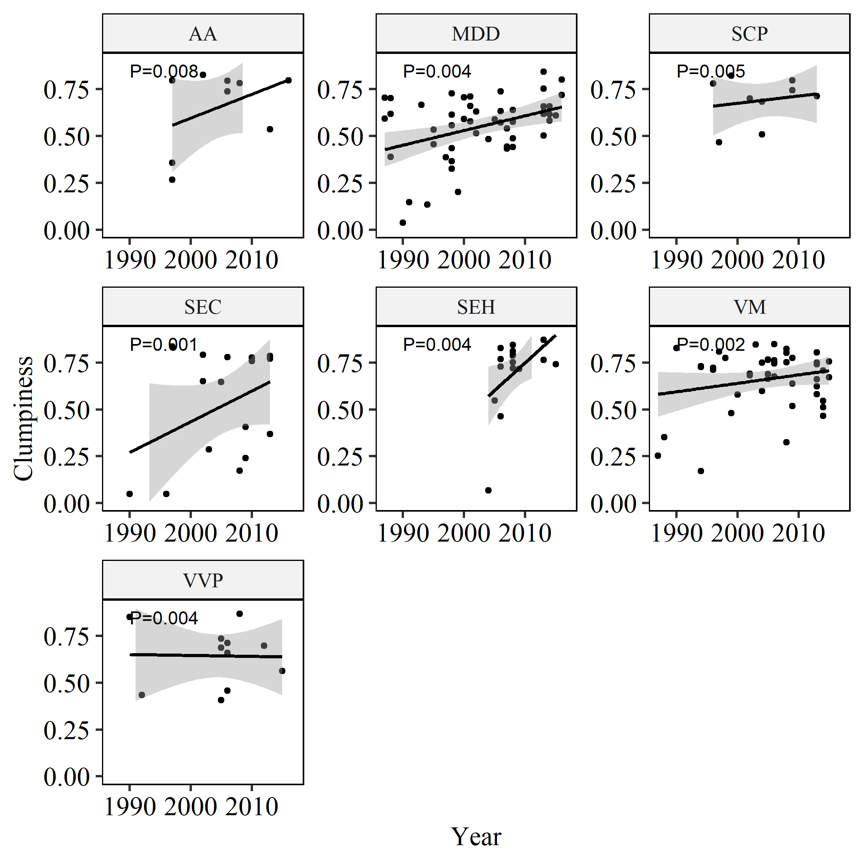
**S10 Fig**. Changes in the clumpiness of high-severity burned area per year from 1987 to 2017 across bioregions. Acronyms follow Table 1.

**
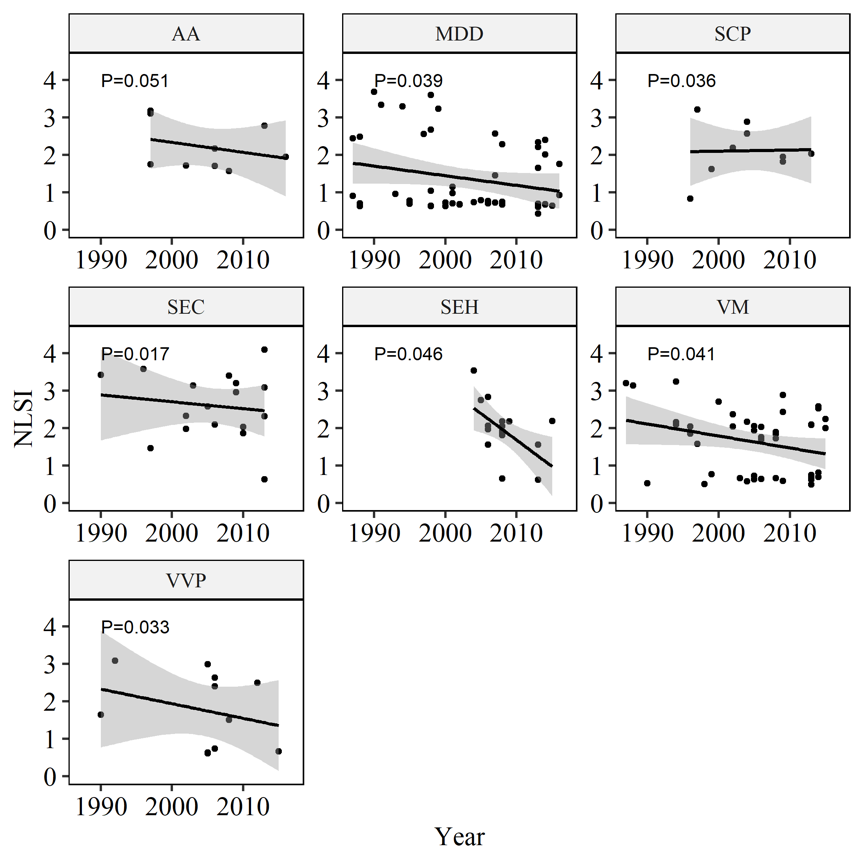
S11 Fig**. Changes in the Normalized Landscape Shape Index (NLSI) of high-severity burned area per year from 1987 to 2017 across bioregions. Acronyms follow Table 1.

Turner, M.G., Gardner, R.H., O'neill, R.V. & O'Neill, R.V. (2001). *Landscape ecology in theory and practice*. Springer.
